# Single‐cell multi‐omics characterises transcriptional and chromatin accessibility heterogeneity of immune cells in allergic rhinitis

**DOI:** 10.1002/ctm2.70854

**Published:** 2026-10-13

**Authors:** Yisha Wu, Zhihan Xu, Shikai Han, Zhongzhen Liu, Rui Dong, Chuanyu Liu, Jianhua Yin, Jinmei Xue

**Affiliations:** ^1^ Department of Otolaryngology, Head and Neck Surgery Second Hospital Shanxi Medical University Taiyuan China; ^2^ Shanxi Key Laboratory of Rapid Diagnosis and Precision Treatment of Airway Allergic Diseases Head & Neck Surgery, Second Hospital, Shanxi Medical University Taiyuan China; ^3^ BGI Collaborative Center for Future Medicine Shanxi Medical University Taiyuan China; ^4^ Shanxi Medical University Taiyuan China; ^5^ State Key Laboratory of Genome and Multi‐omics Technologies BGI Research Shenzhen China

**Keywords:** allergic rhinitis, epigenetic, immune cells, multi‐omics

## Abstract

**Background:**

Allergic rhinitis (AR) remains a global health challenge. However, the transcriptional and epigenetic features associated with seasonal allergic rhinitis (SAR) and perennial allergic rhinitis (PAR) relative to non‐allergic rhinitis (NAR) remain poorly characterised at single‐cell resolution.

**Methods:**

We performed integrated single‐cell transcriptomic and epigenomic profiling of nasal mucosal tissues from 22 patients, including NAR (*n* = 8), SAR (*n* = 6), and PAR (*n* = 8), to characterise transcriptional and epigenetic patterns associated with immune cell heterogeneity.

**Results:**

We identified eight major immune cell types and 29 immune cell subsets across the nasal mucosa. Comparisons with NAR revealed distinct transcriptional and epigenetic features in SAR and PAR across both myeloid and T‐cell compartments. In Macro_CCL3 cells, SAR showed enrichment of inflammation‐associated programs, whereas PAR showed greater enrichment of antigen‐presentation‐related features. CD4_Tcm cells exhibited subtype‐associated epigenetic differences, with IL‐1β/IL‐17‐related features enriched in SAR and eosinophil‐ and Th2‐associated programs enriched in PAR. CD8_Trm cells in both AR groups showed cytotoxicity‐related features, including increased accessibility near *GZMB* and *PRF1*, while distinct inferred regulatory patterns were observed across the two AR groups. In addition, T‐cell co‐expression modules identified by hdWGCNA showed distinct associations with NAR, SAR, and PAR. sLDSC analysis revealed significant enrichment of pollinosis‐associated heritability in CD8 T‐cell open chromatin regions.

**Conclusion:**

Our study characterises distinct immune regulatory features associated with SAR and PAR relative to NAR across innate and adaptive immune compartments, providing a multi‐omic framework for characterising AR heterogeneity and identifying subtype‐associated molecular features for further investigation.

## Graphical abstract

Key Points

1. SAR and PAR showed distinct immune landscapes.

2. Macro_CCL3 showed phenotype‐specific inflammatory and antigen‐presentation features.

3. CD8_Trm displayed subtype‐associated chromatin accessibility linked to cytotoxicity‐related signatures.

4. T‐cell co‐expression modules further characterised immune heterogeneity across AR phenotypes.

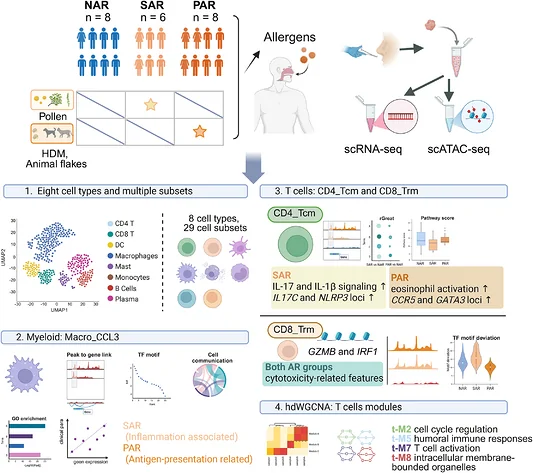

## 1 INTRODUCTION

Allergic rhinitis (AR) is a major global health challenge, with a rising prevalence estimated at 18.1% in China.^1^ Clinically, AR is classified into seasonal allergic rhinitis (SAR) and perennial allergic rhinitis (PAR) according to allergen exposure patterns, which range from intermittent exposure to seasonal pollens (e.g., Artemisia) to continuous exposure to perennial allergens such as house dust mites.^2^, ^3^, ^4^ Although SAR and PAR exhibit distinct temporal symptom profiles, whether these clinical phenotypes are associated with distinct immune regulatory states or shared allergic inflammatory pathways remains incompletely understood.

Single‐cell sequencing has provided new opportunities to characterise cellular heterogeneity and disease‐associated immune states in allergic and inflammatory diseases. Previous single‐cell studies identified disease‐associated immune populations and regulatory networks in allergic skin disease and asthma.^5^, ^6^, ^7^, ^8^ For example, IL‐1β‐induced LY6D^+^ club cells and IDO1^+^ fibroblasts were involved in neutrophil recruitment in patients with nasal polyps,^7^, ^9^ as well as distinct macrophage and T‐cell states in allergic asthma.^10^ In addition, biomarkers such as IgG4 and CD23 were investigated for monitoring responses to sublingual immunotherapy.^11^ Despite these advances, the immune regulatory landscapes associated with AR phenotypes remain poorly characterised, particularly at the integrated transcriptomic and epigenomic levels.

By linking transcriptional profiles with chromatin accessibility, single‐cell multi‐omics can provide complementary information on cellular heterogeneity and potential regulatory programs that may not be fully captured by transcriptomic profiling alone.^12^, ^13^ Such approaches have been increasingly applied across immune‐mediated diseases, cancer, and inflammatory disorders, providing a broader framework for investigating disease heterogeneity and cellular regulatory states.^14^, ^15^, ^16^ However, the transcriptional and epigenetic features associated with SAR and PAR relative to NAR remain poorly characterised.

To address this gap, we performed single‐cell RNA sequencing (scRNA‐seq) and single‐cell assay for transposase‐accessible chromatin using sequencing (scATAC‐seq) on nasal mucosal tissues from patients with non‐allergic rhinitis (NAR), SAR, and PAR, with comparisons focused on SAR versus NAR and PAR versus NAR. By mapping transcriptional states and chromatin accessibility landscapes across innate and adaptive immune compartments, we identified subtype‐associated immune cell signatures. These findings provide a comprehensive resource for characterising immune heterogeneity and identifying molecular features associated with AR phenotypes.

## 2 MATERIALS AND METHODS

### 2.1 Study population and data collection

Samples were collected from patients who attended the Second Hospital of Shanxi Medical University between April 2024 and June 2024. All participants had experienced nasal symptoms for more than three years and underwent surgical treatment for nasal septal deviation or nasal bone fracture. Nasal mucosal samples were collected during surgery for subsequent scRNA‐seq and scATAC‐seq analyses. Patients with comorbid asthma, acute respiratory tract infections, or autoimmune diseases were excluded. Patients who had received systemic corticosteroids, immunosuppressants, or biologic therapies within four weeks of sample collection were also excluded.

A total of 39 samples were initially subjected to sequencing. One sample from a patient with concomitant fungal rhinosinusitis was excluded. Samples with data available from only one omics platform (scRNA‐seq or scATAC‐seq) were excluded. In addition, participants with positive skin prick test (SPT) reactions to only a single allergen at the ‘+’ level (and no other positive reactions) were also excluded from the analysis. Following these exclusion criteria, 22 samples with both scRNA‐seq and scATAC‐seq data were included in the final analysis (Figure S1A).

Based on clinical symptoms and allergic sensitisation profiles, participants were classified into three groups: NAR, SAR, and PAR. NAR was defined as the presence of nasal symptoms but without clinical signs of infection and without evidence of systemic allergic sensitisation, as indicated by negative SPT results.^17^ The diagnosis of SAR and PAR was first required to meet the diagnostic criteria of AR. AR diagnosis was based on typical nasal symptoms and positive SPT reactivity according to established clinical criteria.^18^ Antihistamines were discontinued for at least one week before testing, and reactions were recorded after 15 min. A wheal diameter ≥3 mm or a reaction graded as ‘+’ or higher was considered positive. Based on allergen sensitisation patterns and symptom seasonality, patients with AR were further classified as SAR or PAR.^4^ SAR was defined by sensitisation to seasonal outdoor allergens (willow, elm, artemisia, and poplar) with corresponding seasonal symptoms, whereas PAR was defined by sensitisation to perennial indoor allergens (house dust mites and animal dander) with persistent symptoms.^18^ Written informed consent was obtained from all participants before study entry.

### 2.2 Sample collection and single‐cell sequencing

Fresh tissue samples were preserved immediately in a solution at 4°C and transported quickly to the laboratory.^19^ A tissue digestion solution was prepared using an optimised dissociation protocol for nasal mucosa to produce a single‐cell suspension. This suspension was then filtered through a 40 µm strainer, centrifuged, and resuspended in PBS + .04% BSA, maintaining cell viability at over 85%. The final concentration was adjusted to 1,000–2,500 cells/µL for scRNA‐seq and scATAC‐seq, respectively. Single‐cell sequencing was then performed using the DNBelab C4 platform. The raw sequencing data were processed via a custom pipeline to generate a gene expression matrix (scRNA‐seq) and fragment files (scATAC‐seq) (Tables S2 and S3).

### 2.3 Single‐cell RNA sequencing data analysis

Downstream analysis of single‐cell transcriptomic data was performed using Seurat V4. Initial filtering was performed with min.cells = 3 and min.features = 200. Additional cell‐level quality control was then performed for each library. Cells with nFeature_RNA values outside the 5th–95th percentile range of the corresponding library distribution were excluded to remove cells with abnormally low or high gene counts. Cells with a mitochondrial gene percentage (percent.mt) > 25% and above the 90th percentile of the corresponding library distribution were also excluded to remove cells with excessive mitochondrial contamination. DoubletFinder was used to identify and remove doublets from the single‐cell RNA sequencing data.^20^ Following filtration, the remaining high‐quality cells underwent log‐normalisation and scaling procedures. Highly variable genes were then identified, and principal component analysis (PCA) was performed using the RunPCA function. Sample‐related batch effects were corrected using Harmony.^21^ The RunUMAP was subsequently applied to generate low‐dimensional embeddings for visualising cell distributions. Clusters containing fewer than 100 cells were excluded from downstream analysis. Cell type‐specific marker genes for each cluster were identified using the FindAllMarkers function. Immune cell subpopulations (including T cells, B cells, and myeloid cells) were annotated based on established markers (e.g., CD3D, MS4A1, CD68). After extraction, these subsets underwent unsupervised subclustering using PCA and graph‐based clustering. The clustering resolution was determined based on the desired level of granularity, enabling the identification of finer subpopulations, which were then annotated using established markers.^9^, ^22^

### 2.4 Differential gene expression and pathway analysis

Differentially expressed genes (DEGs) across groups were identified using Seurat's FindMarkers function, with statistical significance defined as FDR < .05 and |log2FC| > .25 (Wilcoxon rank‐sum test). Pathway enrichment analysis was conducted using the R package clusterProfiler (v4.2). Terms with FDR‐adjusted *p* values < .05 were considered significantly enriched. Gene set scores were calculated using the AddModuleScore function. Statistical significance was assessed using the Wilcoxon rank‐sum test with Benjamini–Hochberg (BH) correction.

For the CD14_Mono and Macro_CCL3 subsets, pseudobulk gene expression profiles were generated at the sample level, and differential expression analysis between groups was performed using edgeR. Sex and age were adjusted as covariates. DEGs were identified with a threshold of FDR < .1 and |log2FC| > .25.

For bulk RNA‐seq data (GSE49782,^23^ GSE50223^24^), normalisation was performed using the normalizeBetweenArrays function. Differential expression analysis was conducted using limma (v3.50), applying thresholds of adjusted *p* value < .05 and |log2FC| > 1.

### 2.5 Single‐cell ATAC sequencing data analysis

Fragment files were used as input for downstream analysis with ArchR.^25^ The analysis workflow began with createArrowFiles, using minFrags = 1000 and minTSS = 4 as initial filtering thresholds. For each library, cells with nFrags, NucleosomeRatio, or ReadsInBlacklist values above the respective 95th percentile were subsequently excluded, as were cells with TSS Enrichment > 25. Potential doublets were identified and removed using the addDoubletScore and filterDoublets functions with a filterRatio of 2. All filtering steps were performed independently for each library. Dimensionality reduction was achieved through iterative Latent Semantic Indexing (LSI). Harmony was used to eliminate the batch effects among all samples. Cell clustering was then performed on the reduced dimensions using the addClusters function. Clusters derived from one sample and undefined clusters containing fewer than 100 cells were excluded from downstream analysis.

Major cell types were manually annotated based on marker gene activity scores. For each major immune compartment, including T cells, B cells, and myeloid cells, the corresponding scATAC‐seq cells were extracted and independently subjected to dimensionality reduction and re‐clustering to resolve finer cell subsets. The corresponding scRNA‐seq subset was used as the reference for cross‐modality cell‐type annotation. Constrained label transfer was performed using the scRNA‐seq reference, and the AddGeneIntegrationMatrix function was used to integrate the scRNA‐seq reference with the scATAC‐seq dataset and transfer cell‐type labels across modalities. The transferred annotations were subsequently evaluated against scATAC‐seq‐derived gene activity scores and canonical marker genes to assess the consistency of the predicted cell identities with the underlying chromatin accessibility profiles.

### 2.6 Differential peak analysis and functional enrichment

For differential chromatin accessibility analysis, we conducted peak calling analysis using the addReproduciblePeakSet function and MACS2 algorithm at the group level to identify chromatin accessible regions. The peaks were subsequently integrated into the ArchR project through the addPeakMatrix function.

Differentially accessible regions (DARs) were identified using the getMarkerFeatures with thresholds (FDR < .1 and |log2FC| > .25). Following DARs identification, functional enrichment analysis was performed using the rGREAT (v2.3) to annotate the biological relevance of the identified regions. Terms with FDR‐adjusted *p* values < .05 were considered significantly enriched.

### 2.7 Motif enrichment analysis

We performed transcription factor (TF) motif analysis through the following steps: First, the detected chromatin accessible regions were aligned with known TF binding motifs from the CIS‐BP database. To correct for sequence preference bias, the addDeviationsMatrix function was then employed to calculate deviation scores. Differential motif enrichment analysis was performed across groups using the getMarkerFeatures function. Motifs with a |MeanDiff| > .2 and FDR < .05 were considered differentially enriched between groups (Wilcoxon rank‐sum test).

### 2.8 Peak‐to‐gene linkage analysis

To identify potential regulatory relationships between chromatin accessibility and gene expression, we performed peak‐to‐gene linkage analysis. Using the addPeak2GeneLinks function, we calculated accessibility‐expression correlations between chromatin accessible regions and their associated gene promoters to identify candidate cis‐regulatory elements. High‐confidence linkages were defined as those demonstrating a correlation coefficient > .5 with FDR < .01. These peak‐gene pairs were subsequently visualised to illustrate putative regulatory networks.

### 2.9 Cell–cell interaction analysis

We employed CellChat (v1.5) to characterise intercellular communication networks among distinct cell populations.^26^, ^27^ The analysis was performed independently for the NAR, SAR, and PAR groups to identify condition‐specific interaction patterns. The communication interactions involving fewer than 10 cells were filtered out. The netVisual function was utilised to visualise ligand‐receptor‐mediated cell interactions.

### 2.10 Gene regulatory network inference

Transcription factor regulatory networks within specific cell subpopulations were inferred using the pySCENIC pipeline.^28^ The raw count matrix was used as input for GRNBoost2 to infer TF‐target gene co‐expression relationships. Subsequently, identify enriched cis‐regulatory motifs within the initially inferred co‐expression modules were identified and candidate TF‐target gene regulatory relationships were refined. AUCell was then applied to calculate regulon activity scores at the single‐cell level. Finally, regulon specificity scores (RSS) were calculated to evaluate the cell‐type specificity of individual regulons.

### 2.11 High‐dimensional WGCNA

We performed high‐dimensional weighted gene co‐expression network analysis (hdWGCNA) on T‐cell subsets using R package hdWGCNA.^29^ To reduce background noise, genes detected in fewer than 5% of cells were excluded from the analysis. Metacells were constructed using a k‐nearest‐neighbour graph with k = 25 to reduce technical noise and improve the robustness of gene co‐expression estimates. During network construction, a soft threshold of 8 was selected based on scale‐free topology fitting (*R* ^2^ = .87), and a signed hybrid network was constructed to preserve the direction of gene–gene correlations. Gene dendrograms were visualised using the PlotDendro function, with modules defined through dynamic tree cutting and module merging. Module eigengenes (t‐Ms) were then correlated with cellular clusters to identify modules associated with specific cell populations. The top 100 genes within target modules were designated as hub genes, followed by Gene Ontology (GO) enrichment analysis using the clusterProfiler package.

### 2.12 Linkage disequilibrium score regression

To quantify cell type‐specific heritability enrichment, we applied stratified Linkage Disequilibrium Score Regression (sLDSC).^30^, ^31^ Summary statistics for six allergy‐ and airway‐related phenotypes, including AR, pollinosis, allergic conjunctivitis, asthma, chronic sinusitis, and nasal polyps, were obtained from the BioBank Japan (BBJ) database. Cell type‐specific chromatin accessibility peaks were identified using scATAC‐seq data. The genome coordinates were then converted to the hg19 reference using UCSC LiftOver, with analysis windows spanning ± 1.0 kb centred on each peak. The 1000 Genomes Project Phase 3 East Asian (EAS) reference panel was used as the LD reference. Significant heritability correlations were determined at FDR threshold of .05.

## 3 RESULTS

### 3.1 A comprehensive immune cell atlas of AR

To investigate the cellular and molecular landscapes associated with AR phenotypes, nasal mucosal tissues were collected. The samples were stratified into three groups according to allergen sensitisation profiles: NAR (*n* = 8), SAR (*n* = 6), and PAR (*n* = 8) (Tables 1 and S1). The study was designed to characterise heterogeneity in immune cells, and downstream analyses were restricted to immune cell populations. After quality control, a total of 94,195 and 61,918 immune cells were retained for scRNA‐seq and scATAC‐seq analysis, respectively. We identified eight major immune cell types and 29 immune cell subsets by scRNA‐seq, and 22 immune cell subsets by scATAC‐seq (Figures 1A and B and S2).

**TABLE 1:** Demographic data of the study groups.

sample	Age	Sex	VAS_score	Total_IgE	EOS_Count	Group
AR04	33	Female	16	101.34	.09	PAR
AR05	35	Male	26	82.18	.19	PAR
AR07	29	Male	26	500	.22	PAR
AR09	60	Female	0	53.94	.04	NAR
AR10	14	Male	0	14.8	.04	NAR
AR14	35	Female	24	79.81	.09	PAR
AR17	19	Male	4	44.65	.12	NAR
AR19	39	Male	41	77.68	.27	SAR
AR20	26	Male	1	41.75	.04	NAR
AR21	35	Male	22	251.92	.08	PAR
AR22	47	Male	0	78.98	.09	NAR
AR23	24	Male	5	74.84	.09	NAR
AR24	38	Female	23	81.44	.12	SAR
AR25	17	Male	14	187.37	.29	PAR
AR26	25	Male	31	200.42	.04	PAR
AR27	35	Female	22	98	.15	PAR
AR30	35	Male	26	172.97	.41	SAR
AR31	18	Male	5	76.92	.1	NAR
AR33	40	Male	7	42.9	.13	NAR
AR34	33	Male	12	110.24	.07	SAR
AR35	64	Male	29	226.56	1.19	SAR
AR36	38	Female	40	90.19	.2	SAR

Abbreviations: NAR, non‐allergic rhinitis; PAR, perennial allergic rhinitis; SAR, seasonal allergic rhinitis.

**FIGURE 1:**
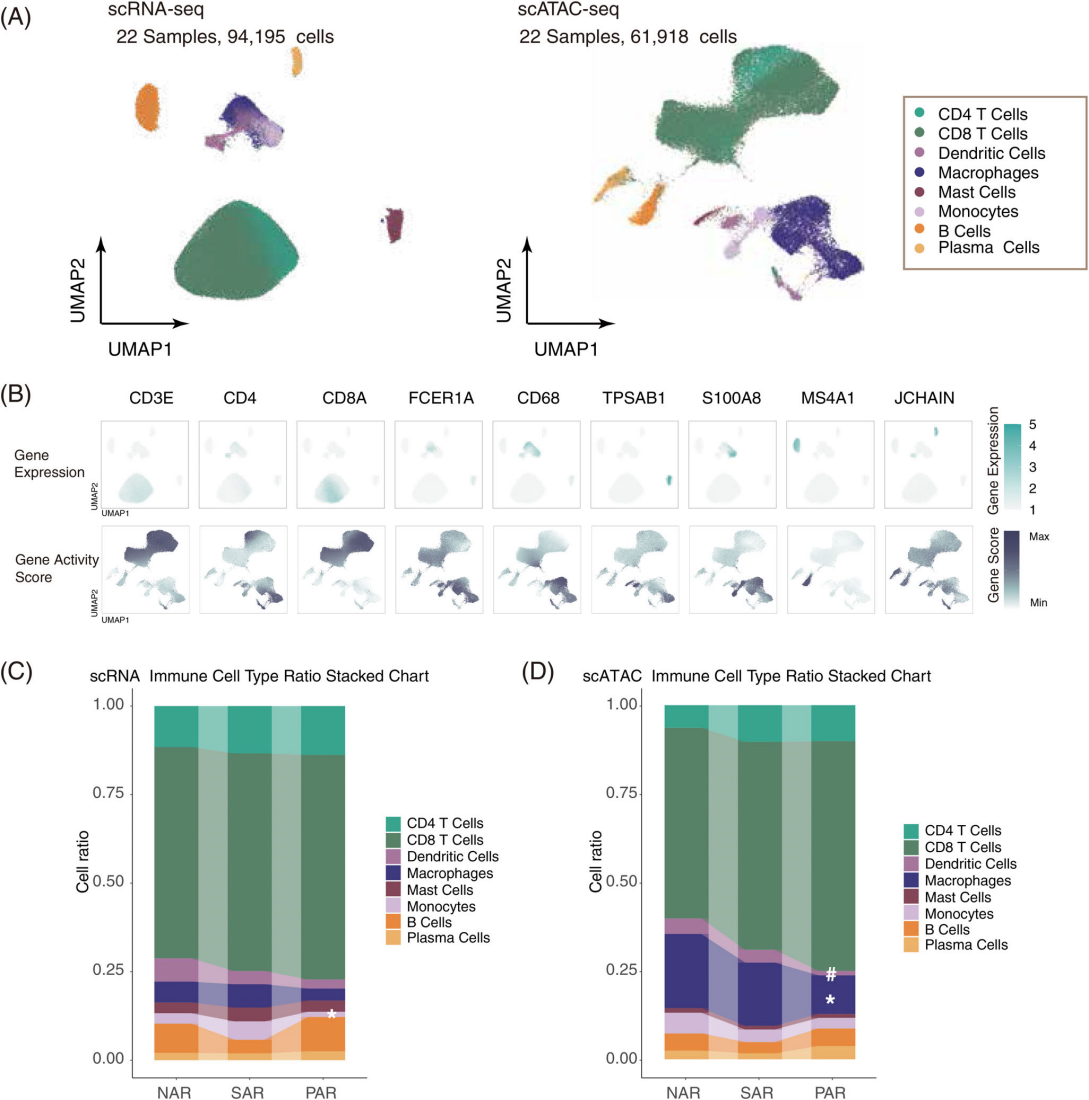
Single‐cell multi‐omics profiling of immune cells in allergic rhinitis (AR) patients. (A) Uniform Manifold Approximation and Projection (UMAP) visualisation of 156,113 immune cells from 22 patients across the scRNA‐seq and scATAC‐seq datasets, coloured by annotated cell types. (B) Feature plots showing the expression of selected marker genes in scRNA‐seq and gene activity scores derived from chromatin accessibility in scATAC‐seq across major cell types. (C, D) Cell‐type composition across disease groups in the scRNA‐seq (C) and scATAC‐seq (D) datasets. * indicates comparison with NAR, *p* < .05; # indicates comparison with SAR, *p* < .05. Data in (C) and (D) were analysed using the Wilcoxon rank‐sum test.

Analysis of cellular composition revealed notable differences in myeloid cell populations across groups in both scRNA‐seq and scATAC‐seq data (Figures 1C and D and S3). Specifically, scRNA‐seq analysis showed a significantly lower proportion of monocytes in PAR than NAR. scATAC‐seq analysis revealed a reduced proportion of macrophages in PAR compared to NAR. The proportion of dendritic cells was decreased in the PAR group compared with the SAR group. No significant differences in the proportions of other immune cell subsets were detected among the groups.

### 3.2 Distinct myeloid regulatory patterns in SAR and PAR

Next, we characterised myeloid cell heterogeneity and identified 10 transcriptionally distinct subsets (eight subsets from scATAC‐seq data). These subsets comprised five macrophage subsets, classical monocytes (CD14_Mono), conventional dendritic cells (cDCs), plasmacytoid DCs (pDCs), migratory DCs (migDCs), and mast cells (Figures 2A and S4A and B). Although the proportions of these subsets remained relatively stable across groups, distinct transcriptional and epigenetic differences were observed in specific populations (Figures S4C and 2B).

**FIGURE 2:**
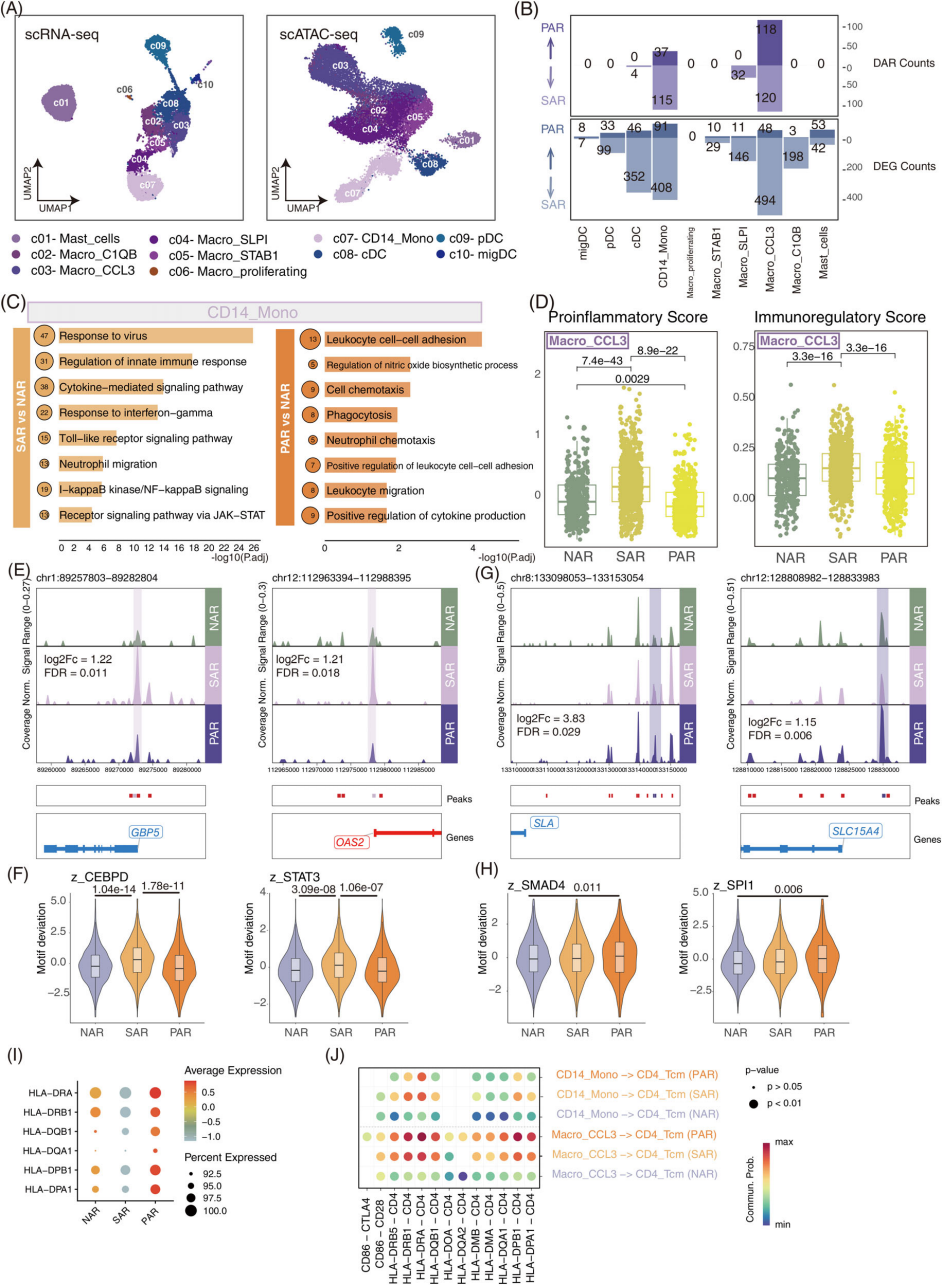
Multi‐omic characterisation of myeloid cell heterogeneity. (A) UMAP visualisation of myeloid cell subsets from scRNA‐seq (Left) and scATAC‐seq (Right) data. (B) Differentially accessible regions (DARs) in SAR/PAR versus NAR groups (Top, PAR: dark purple; SAR: light purple). Number of differentially expressed genes (DEGs) in SAR/PAR versus NAR groups (Bottom, PAR: dark blue; SAR: light blue). (C) Pathway enrichment analysis of DEGs in CD14_Mono for SAR vs. NAR and PAR vs. NAR comparisons. (D) Box plots comparing pathway activity scores for proinflammatory and immunoregulatory programs across the NAR, SAR, and PAR groups in Macro_CCL3, with adjusted *p* values displayed above each comparison. Data were analysed by Wilcoxon rank‐sum test. (E) Genome browser tracks displaying DARs in Macro_CCL3. The peaks panel highlights the different significant peaks in SAR vs. NAR. (F) Violin plots of motif deviation for CEBPD and STAT3 across NAR, SAR, and PAR groups. The motifs that show significant differences in the SAR group are presented. FDR values from Wilcoxon rank‐sum test. (G) Genome browser tracks displaying DARs in Macro_CCL3. The peaks panel highlights the different significant peaks identified by ArchR in PAR vs. NAR. (H) Violin plots of motif deviation scores for SMAD4 and SPI1 across NAR, SAR, and PAR groups. The motifs that show significant differences in the PAR group are presented. FDR values from Wilcoxon rank‐sum test. (I) Dot plot showing the expression levels of HLA‐II genes among the different groups in Macro_CCL3. There are significant differences between the PAR and SAR groups. (J) Ligand‐receptor pairs grouped by antigen presentation signalling pathways are displayed for CD14_Mono and Macro_CCL3.

Notably, CD14_Mono and Macro_CCL3 showed the most prominent alterations in both SAR and PAR compared with NAR, and were selected for further characterisation (Figures 2B and S4D). In SAR, GO analysis revealed significant enrichment of interferon‐γ (IFN‐γ) response and TLR signalling pathways in CD14_Mono (Figure 2C). Chromatin accessibility analysis showed increased accessibility at *CXCL8* and *CD83* loci in SAR (Figure S4E), which have been associated with inflammatory responses in monocytes.^32^, ^33^ CD14_Mono in PAR showed enrichment of pathways related to leukocyte adhesion, nitric oxide biosynthesis, and neutrophil chemotaxis (Figure 2C). We performed motif enrichment analysis, which showed enrichment of motifs for TFs including CEBPZ, HMGA2, and ARID3C (Figure S4F and G).^34^ Given that the members of the C/EBP family are involved in myeloid cell differentiation and function, we further examined CEBPB, which was associated with both shared and cell type‐specific gene programs in our analysis (Figure S4H).^35^ Among the CEBPB target genes, *ACSL1* loci showed higher chromatin accessibility in both CD14_Mono and Macro_CCL3 (Figure S4I). *ACSL1* has been reported to regulate COX2‐related inflammatory signalling in monocytes and macrophages.^36^ *ACSL1* expression was significantly elevated in SAR compared with NAR, whereas no significant difference was observed between PAR and NAR (Figure S4J).

### 3.3 Distinct regulatory features of Macro_CCL3 in SAR and PAR

Our analysis revealed distinct transcriptional and epigenetic profiles of Macro_CCL3 cells in SAR and PAR compared with NAR. In SAR, Macro_CCL3 cells exhibited a stronger inflammatory and immunoregulatory signature than those in the other groups (Figure 2D). Increased chromatin accessibility was observed at the loci of inflammation‐related genes (*GBP5* and *OAS2*) (Figure 2E). These subsets also showed significantly higher expression of IFN response‐related genes and TNF production‐related genes (Figure S5A–C). Given that IFN signalling has been implicated in myeloid cell activation by modulating chromatin accessibility at binding sites for TFs such as IRF1, CEBP, and AP‐1,^37^ we performed motif enrichment analysis to identify TFs. We identified that motifs associated with CEBPD^38^ and STAT3^39^ were enriched in SAR (Figures 2F and S5D). Compared with NAR, IFN‐inducible chemokines such as *CXCL9*, *CXCL10*, and *CXCL11* were significantly more highly expressed in SAR (Figure S5E and F). Furthermore, SAR patients showed enhanced predicted CXCL8/9/10‐associated communication patterns involving myeloid cells (Figure S5G and H).

In comparison, Macro_CCL3 cells in PAR patients were characterised by the enrichment of antigen presentation and immunoglobulin‐mediated immune response pathways (Figure S6A and B). We observed increased chromatin accessibility at antigen‐presentation genes *SLA* and *SLC15A4* loci of PAR, along with increased motif activity for SPI1 and SMAD4 (Figures 2G and H and S6C). Compared to SAR, the HLA class II genes such as *HLA‐DPA1*, *HLA‐DPB1*, and *HLA‐DQA1* were significantly upregulated in the PAR group (Figures 2I and S6E). Cell–cell communication analysis further revealed stronger HLA class II‐associated communication patterns between PAR macrophages and CD4 T cells, particularly CD4 central memory T cells (CD4_Tcm) (Figures 2J and S6D). Notably, *HLA‐DQA2* was expressed at significantly higher levels in the PAR than NAR group (Figure S6F and G).^40^, ^41^ Peak‐to‐gene linkage analysis showed the associations between accessible chromatin regions and *HLA‐DQA2* expression in Macro_CCL3 cells, accompanied by increased accessibility at corresponding chromatin regions (Figure S6H). Although *HLA‐DQA2* expression was not correlated with VAS scores or blood eosinophil counts, it showed a positive correlation with total serum IgE levels (Figure S6I).

### 3.4 Epigenetic alterations in CD4_Tcm in AR

A hallmark of adaptive immune responses in AR is the presentation of allergens to CD4 T cells, which triggers Th2 differentiation and initiates downstream inflammatory cascades.^42^, ^43^ We re‐clustered the T‐cell populations into 12 distinct subsets (nine subsets identified from scATAC‐seq data) (Figures 3A and S7A–C). The cellular composition across groups was summarised (Figure S7D). Next, we analysed DEGs and DARs. Although CD4_Tcm and CD8 tissue‐resident memory T cells (CD8_Trm) exhibited limited transcriptomic differences compared with NAR, they displayed distinct epigenetic differences (Figure 3B).

**FIGURE 3:**
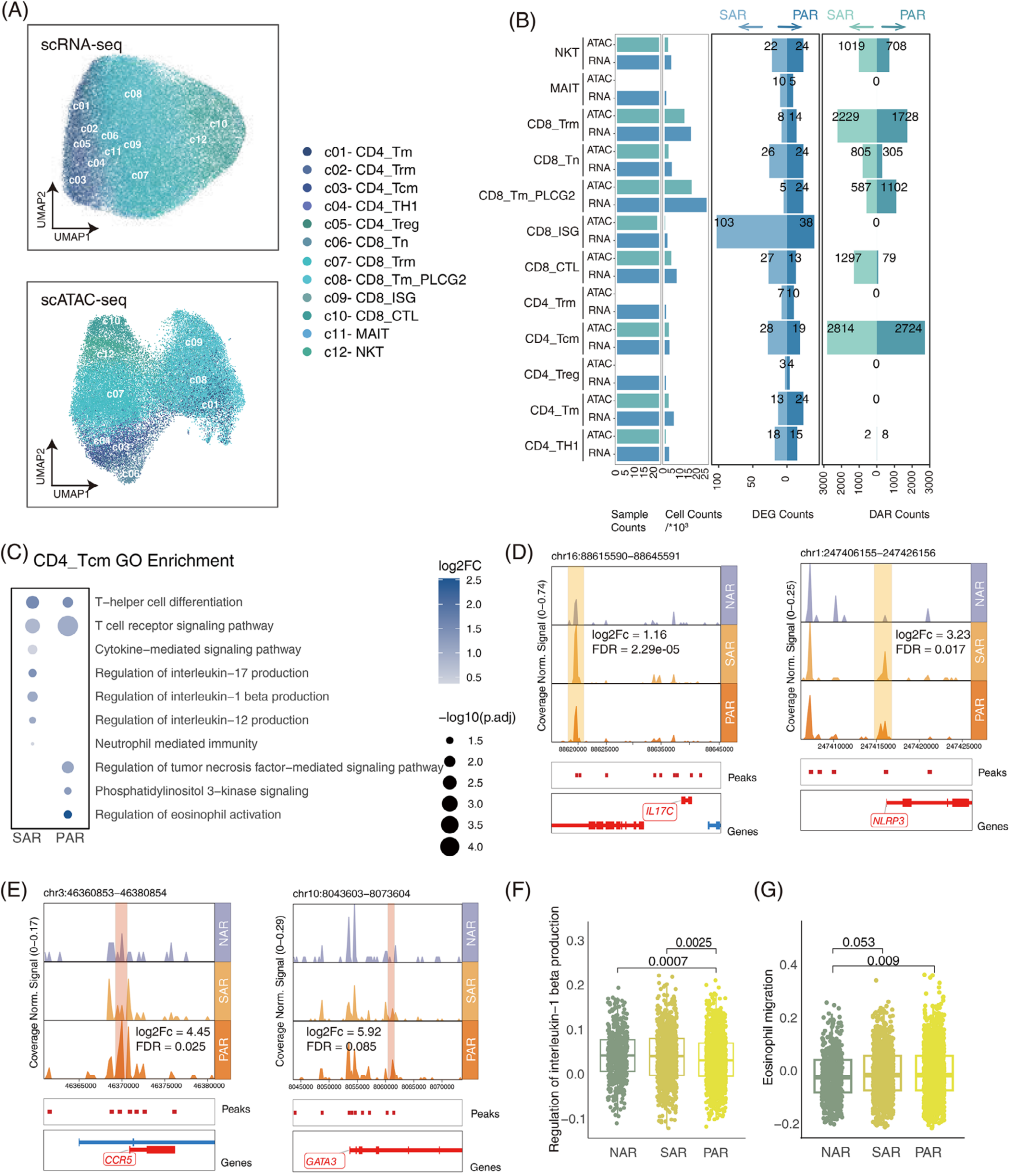
Integrated analysis of T‐cell transcriptomic and epigenomic features across disease states. (A) UMAP plots displaying T‐cell subsets from scRNA‐seq (TOP) and scATAC‐seq (Bottom) data. (B) Comparative analysis of RNA/ATAC data, left panel showing cell numbers per cell subset, middle displaying DEGs counts (SAR/PAR vs. NAR), right panel showing DARs counts (PAR/SAR vs. NAR), with arrow direction indicating PAR/SAR group vs. NAR. Statistical comparisons were performed using Wilcoxon rank‐sum tests, and *p* values were adjusted for multiple testing using the Benjamini–Hochberg (BH) method. (C) Enrichment analysis of upregulated DARs compared to the NAR group in CD4_Tcm. (D) Genome tracks showing DARs in CD4_Tcm with SAR‐specific significant peaks. The peaks panel highlights the different significant peaks identified by ArchR in SAR vs. NAR. (E) Genome tracks displaying DARs in CD4_Tcm with PAR‐specific significant peaks. The peaks panel highlights the different significant peaks identified by ArchR in PAR vs. NAR. (F, G) Box plots comparing pathway activity scores for IL1β signalling (F) and eosinophil migration (G) across the NAR, SAR, and PAR groups, with adjusted *p* values shown above comparisons. Data were analysed by Wilcoxon rank‐sum test.

In CD4_Tcm, pathways related to helper T‐cell differentiation and the T‐cell receptor signalling pathway were enriched in the SAR and PAR groups (Figure 3C). However, their inflammatory profiles were distinct. The SAR group showed enrichment for IL‐17 and IL‐1β signalling pathways (Figure 3C and F). Concurrently, increased accessibility was observed at inflammation‐related loci (*IL17C* and *NLRP3*) in SAR patients (Figure 3D).^24^, ^44^ IL‐1β elevation was associated with T‐cell activation‐related responses and cytokine production during AR progression.^45^, ^46^ Moreover, serum IL‐17 levels have been reported to correlate with clinical parameters in AR patients during the pollen season.^47^ Analysis of bulk RNA‐seq datasets from allergen‐challenged tissues and peripheral blood further supported IL‐1β/IL‐17‐associated transcriptional programs in the SAR cohort (Figure S7E and F). PAR patients displayed a distinct immune signature characterised by enrichment of eosinophil activation and phosphatidylinositol 3‐kinase signalling (Figure 3C). Chromatin accessibility was significantly increased at key eosinophil activation gene loci, such as *CCR5* and *GATA3* (Figure 3E). Pathway analysis further showed enrichment of eosinophil migration pathways in PAR (Figure 3G).

TF motif analysis of CD4_Tcm revealed enrichment of the AP‐1 family in both SAR and PAR (Figure S7G). These patterns were observed in the inferred regulatory networks (Figure S7H). RARA and RARG motif accessibility in SAR were significantly increased; PAR showed increased IRF4 and SMAD4 motif accessibility in CD4_Tcm.

### 3.5 CD8_Trm epigenetic alterations are associated with cytotoxic signatures in AR

Tissue‐resident memory T cells are localised at mucosal barriers and contribute to site‐specific immune surveillance.^48^ To characterise their potential involvement in AR phenotypes, we examined transcriptional and epigenetic features of CD8_Trm cells. SAR patients showed enrichment of the leukocyte chemotaxis pathway, whereas PAR patients showed enrichment of a pathway related to positive regulation of T‐cell migration and IL‐2 signalling (Figure 4A). CD8_Trm cells from both SAR and PAR showed enrichment of IFN‐γ signalling and immunological synapse‐related pathways (Figures 4A and S8A–C). IL‐2 is known to enhance CD8 T‐cell effector functions,^49^ and genomic studies have previously linked the IL2/IL21‐containing 4q27 locus to AR susceptibility.^50^ Our analysis revealed increased chromatin accessibility at the *IL21R* promoter region in both AR groups, accompanied by stronger peak‐to‐gene associations (Figure 4B and C). Given the reported role of IL21 in cytotoxic T‐cell differentiation,^51^, ^52^ we further evaluated cytotoxicity‐related features in CD8_Trm cells. Notably, chromatin accessibility was significantly increased at regions near cytotoxicity‐associated genes (*GZMB* and *PRF1*), and cytotoxicity scores were higher in both SAR and PAR groups (Figure 4D and E). We identified 26 TFs with differential regulon activity in AR, including RUNX3 (Figure 4F and G). RUNX3 has been reported to be associated with cytotoxic T‐cell differentiation and T‐cell functional states.^53^, ^54^ The PAR group exhibited a distinct NF‐κB‐associated pattern, characterised by enrichment of RELA, REL, and NFKB1 motifs and footprints compared with the SAR group (Figures 4H and S8D and E). Regulatory network analysis showed higher inferred activity of NF‐κB family members (RELA, RELB, and NFKB2) in PAR (Figure 4I).

**FIGURE 4:**
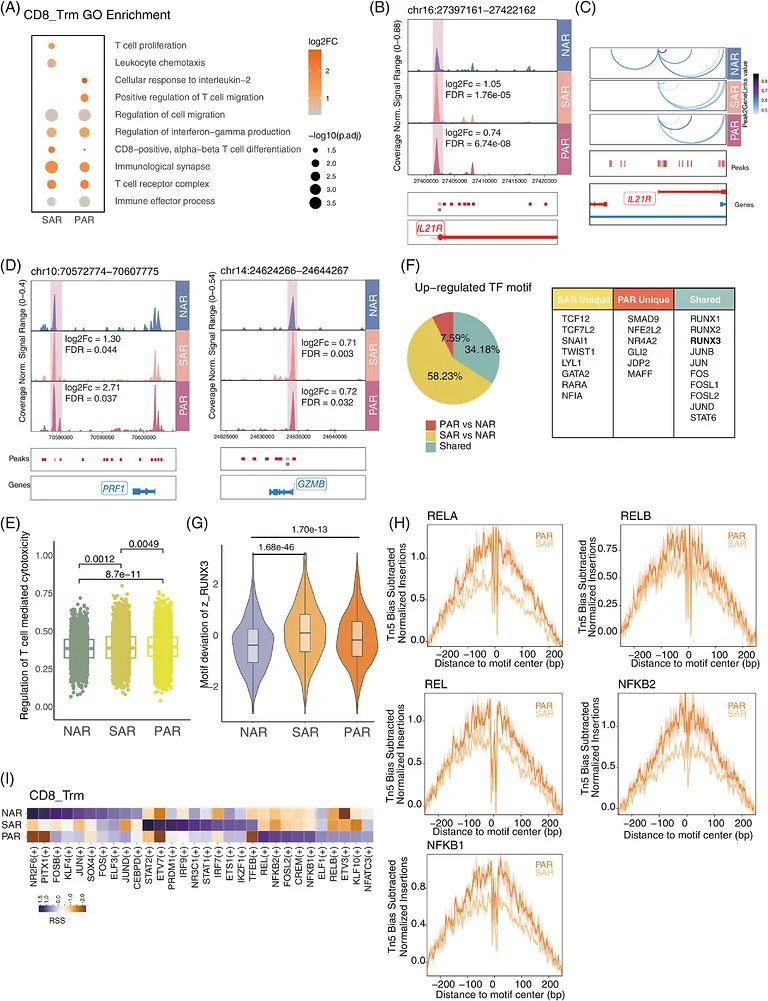
Epigenomic and functional characterisation of CD8 T cells. (A) Enrichment results of upregulated DARs compared to the NAR group in CD8_Trm. (B) Genome tracks showing IL‐21R region in CD8_Trm, highlighting regions simultaneously upregulated in both SAR versus NAR and PAR versus NAR comparisons. (C) Peak‐to‐gene links visualisation of IL21R for each group. (D) Genome tracks showing cytotoxicity genes in CD8_Trm, highlighting regions upregulated in both SAR and PAR groups. (E) Box plots displaying T‐cell‐mediated cytotoxicity pathway scores across groups, annotated with corresponding adjusted *p* values. Data were analysed by Wilcoxon rank‐sum test. (F) Pie chart depicting proportions of upregulated transcription factor motifs (left) with detailed motif annotations (right). (G) Violin plots displaying motif deviation of RUNX3 in NAR, SAR, and PAR groups. Data were analysed by Wilcoxon rank‐sum test. FDR‐adjusted *p* values are shown. (H) Representation of the Tn5 bias‐adjusted TF footprints for candidate TF regulators (NF‐κB‐related motifs) identified by the enrichment motif analysis (light orange: SAR group, dark orange: PAR group). (I) Heatmap visualising the top 10 regulons by normalised RSS in CD8_Trm cells across groups.

To assess whether open chromatin regions were enriched for heritability associated with allergic and nasal diseases, we performed sLDSC using summary statistics from the BBJ database. Using scATAC‐seq peaks extended by ± 1 kb as genomic annotations, we assessed their heritability enrichment relative to a baseline model. Significant enrichment was observed specifically in CD8 T cells for pollinosis, no other significant enrichments were detected (Figure S9).

### 3.6 T‐cell co‐expression modules associated with AR subtypes

Comparison of clinical parameters showed differences between AR and NAR, whereas these parameters showed limited discrimination between AR phenotypes (Figure S10A). To identify molecular features associated with AR phenotypes, we performed hdWGCNA on T cells. This analysis identified eight co‐expression modules, among which four modules (t‐M2, t‐M5, t‐M7, and t‐M8) showed the strongest associations with the above cell subsets (Figures 5A and S10B–D). GO enrichment showed distinct biological characteristics of these modules: t‐M2 was enriched for cell cycle regulation and inflammatory pathways; t‐M5 featured genes involved in humoral immune responses; t‐M7 contained genes encoding chemokines and inflammatory mediators, and was enriched for terms related to T‐cell activation, IFN‐γ signalling, and eosinophil chemotaxis, while t‐M8 was associated with intracellular membrane‐bounded organelles (Figure S10E).

**FIGURE 5:**
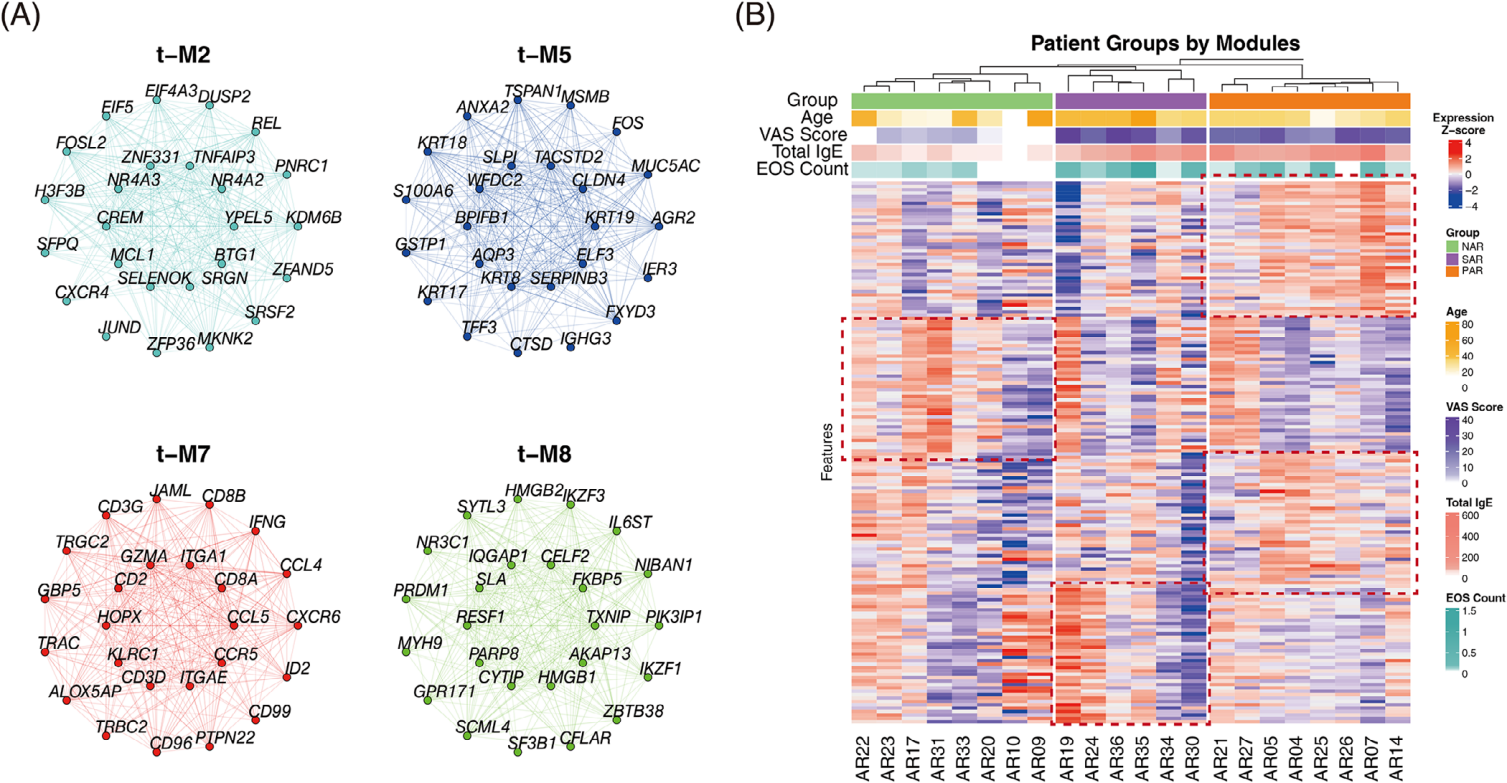
Co‐expression network analysis of gene modules across patient groups. (A) Co‐expression patterns of modules, including t‐M2, t‐M5, t‐M7, and t‐M8. (B) Heatmap displaying cluster patterns of module signatures across different patient groups.

Cluster analysis revealed distinct module expression patterns across disease groups. t‐M5 showed higher module scores in NAR, t‐M8 showed higher module scores in SAR, and t‐M2 and t‐M7 showed higher module scores in PAR (Figure 5B). These modules were also significantly associated with clinical parameters (Figure S10F).

## 4 DISCUSSION

Allergic rhinitis is characterised by significant immunological heterogeneity, and the persistence of the disease is associated with coordinated changes among immune cells.^55^, ^56^, ^57^ In this study, by integrating single‐cell transcriptomic and epigenomic profiling, we identified distinct immune features associated with SAR and PAR relative to NAR across both innate and adaptive immune compartments. Although the overall composition of major immune cell populations remained relatively stable, substantial transcriptional and epigenetic differences were observed across AR phenotypes. SAR showed inflammatory programs involving myeloid and T cells, whereas PAR exhibited stronger antigen‐presentation, Th2‐associated, and NF‐κB‐related features. Thus, SAR and PAR are associated with distinct immune features despite broadly similar cellular compositions.^58^

With the rapid development of high‐throughput technologies, diverse omics approaches, including transcriptomics, genomics, epigenomics, single‐cell and spatial transcriptomics, metabolomics, and microbiome profiling, have been increasingly applied to investigate complex diseases.^59^ Integrating multiple omics layers provides complementary insights into disease‐associated molecular programs and their relationships with clinical phenotypes. For example, studies of inflammatory bowel disease have highlighted the influence of spatial organisation on the gut microbiome, while biologically defined endotypes in chronic obstructive pulmonary disease may provide greater predictive value for treatment responses.^60^, ^61^ In AR, characterisation of the local mucosal microenvironment has facilitated the identification of potential disease‐associated molecular targets.^62^

Among innate immune populations, Macro_CCL3 displayed subtype‐associated molecular signatures. The role of macrophages in nasal diseases, including nasal polyps, has also been increasingly recognised, with studies implicating ALOX15^+^ macrophages and platelet‐derived 5‐hydroxytryptamine in T2 inflammation.^63^, ^64^ Our study showed that SAR Macro_CCL3 cells had a pro‐inflammatory transcriptional signature, with elevated expression of T‐cell recruitment chemokines (*CXCL9*, *CXCL10*, and *CXCL11*), whereas PAR Macro_CCL3 cells displayed antigen‐presentation patterns with increased chromatin accessibility at *HLA‐DQA2* regions. *HLA‐DQA2* was also significantly associated with total IgE levels. Together with its role in antigen presentation, this finding suggests that *HLA‐DQA2* may be relevant to immune stratification, although its relationship with allergic inflammation remains to be determined. These findings raise the possibility that *HLA‐DQA2* may be a candidate for future investigation; however, its biological relevance, feasibility as a therapeutic target, and clinical value require validation in functional and clinical studies. Tissue‐resident macrophages participate in antigen presentation and local immune regulation in allergic airway diseases.^65^, ^66^ These divergent molecular states further characterise the molecular heterogeneity of tissue‐resident macrophages across AR subtypes.

Commensal viruses have been implicated in type I interferon responses in the nasal mucosa during AR, while Trm cells provide local antiviral surveillance and can rapidly respond to antigen re‐exposure.^67^, ^68^, ^69^ Against this broader inflammatory background, we further examined CD8 T‐cell heterogeneity in AR. CD8_Trm cells in our study exhibited increased chromatin accessibility at cytotoxicity‐associated genes, including *GZMB* and *PRF1*, together with higher cytotoxicity scores. Previous studies have reported an expansion of GZMK+CD8 T cells in nasal polyps that was associated with a pro‐inflammatory fibroblast phenotype and neutrophil‐related responses, while *GZMK* expression by CD8 T cells has also been implicated in the early recruitment of eosinophils to the airway in a C3‐dependent context.^70^, ^71^ In addition, allergen sensitisation in the lung has been reported to affect the formation of tissue‐resident CD8 T cells and their antiviral responses.^72^ In our study, SAR showed greater enrichment of RUNX3‐associated activity, whereas PAR exhibited stronger NF‐κB motif enrichment. Given the established involvement of NF‐κB signalling in inflammatory responses, the enrichment of NF‐κB‐related regulatory features in PAR identified NF‐κB‐associated signalling as a candidate pathway for further investigation in PAR.^73^, ^74^ However, whether modulation of NF‐κB signalling has therapeutic potential in PAR remains to be determined through functional and clinical studies. The T‐cell co‐expression modules further characterised differences in immune regulatory programs across AR phenotypes. Importantly, these associations do not establish causal relationships but instead identify candidate regulatory features that warrant further investigation in the context of AR heterogeneity.

Our study has several limitations. First, participants were recruited from a single centre and were undergoing surgery for nasal septal deviation or nasal bone fracture. Multicentre studies involving broader patient populations are needed to improve the generalisability of our findings. Second, our control cohort consisted of non‐allergic surgical patients rather than completely healthy individuals; thus, the identified molecular profiles reflect differences relative to a non‐allergic disease control state rather than an absolute healthy baseline. Third, donor‐level pseudobulk methods and covariate adjustment were not systematically applied across all single‐cell differential analyses, which may introduce potential sample‐level confounding or increase the risk of false‐positive findings. Fourth, scRNA‐seq and scATAC‐seq identify candidate transcriptional and epigenetic associations but do not establish their functional effects or causal relationships. Finally, quantitative individual allergen exposure was not assessed. In addition, nasal allergen provocation testing was not performed, and local allergic rhinitis could therefore not be specifically assessed or excluded.

## 5 CONCLUSIONS

Our study identifies distinct immune regulatory features associated with SAR and PAR relative to NAR and provides a multi‐omic framework for characterising AR heterogeneity and generating hypotheses regarding subtype‐associated molecular features for further investigation.

## AUTHOR CONTRIBUTIONS

*Conceptualisation*: J.X. and J.Y. *Methodology*: Y.W. *Investigation*: Z.X. and Z.L. *Resources*: S.H. and R.D. *Formal analysis*: Y.W. *Writing – original draft*: Y.W. *Writing – review, and editing*: Z.X., S.H., C.L., and R.D. *Supervision*: C.L., J.Y., and J.X.

## CONFLICT OF INTEREST STATEMENT

The authors declare no competing interests.

## ETHICS STATEMENT

This study was conducted in accordance with the Declaration of Helsinki and approved by the Ethics Review Committee of the Second Hospital of Shanxi Medical University (approval number: [2024] YX No.045). Informed consent was obtained from the patient prior to enrolment.

## Supporting information



Supporting information

Supporting information

## Data Availability

All data from this research have been deposited to GSA‐Human, including raw sequencing data (scRNA‐seq FASTQ files, scATAC‐seq FASTQ files) (GSA‐Human: HRA011954, HRA011956). All code used in this study are publicly available at https://github.com/Wuyisha/Allergic‐rhinitis–immunity.

## References

[1] Savouré M , Bousquet J , Jaakkola JJK , Jaakkola MS , Jacquemin B , Nadif R . Worldwide prevalence of rhinitis in adults: a review of definitions and temporal evolution. Clin Transl Allergy. 2022;12:e12130. 10.1002/clt2.12130 PMC8967272 35344304

[2] Zhao Z , Chen L , Huang C , et al. Allergen sensitization patterns in children with allergic rhinitis: insights from a four‐year retrospective study in Shenzhen, China. BMC Pediatrics. 2025;25:544. 10.1186/s12887-025-05885-1 PMC12243126 40634890

[3] Lin H , Lin R , Li N . Sensitization rates for various allergens in children with allergic Rhinitis in Qingdao, China. Int J Environ Res Public Health. 2015;12:10984‐10994. 10.3390/ijerph120910984 PMC4586656 26371014

[4] Ciprandi G , Cirillo I , Vizzaccaro A , et al. Seasonal and perennial allergic rhinitis: is this classification adherent to real life? Allergy. 2005;60:882‐887. 10.1111/j.1398-9995.2005.00602.x 15932377

[5] He H , Suryawanshi H , Morozov P , et al. Single‐cell transcriptome analysis of human skin identifies novel fibroblast subpopulation and enrichment of immune subsets in atopic dermatitis. J Allergy Clin Immunol. 2020;145:1615‐1628. 10.1016/j.jaci.2020.01.042 32035984

[6] Seumois G , Ramírez‐Suástegui C , Schmiedel BJ , et al. Single‐cell transcriptomic analysis of allergen‐specific T cells in allergy and asthma. Sci Immunol. 2020;5:eaba6087. 10.1126/sciimmunol.aba6087 PMC7372639 32532832

[7] Nishide M , Shimagami H , Kumanogoh A . Single‐cell analysis in rheumatic and allergic diseases: insights for clinical practice. Nat Rev Immunol. 2024;24:781‐797. 10.1038/s41577-024-01043-3 38914790

[8] Mitamura Y , Akdis CA . Single‐cell analysis of allergic diseases. Allergy. 2023;78:346‐348. 10.1111/all.15581 36707981

[9] Xie X , Wang P , Jin M , et al. IL‐1β‐induced epithelial cell and fibroblast transdifferentiation promotes neutrophil recruitment in chronic rhinosinusitis with nasal polyps. Nat Commun. 2024;15:9101. 10.1038/s41467-024-53307-0 PMC11496833 39438439

[10] Alladina J , Smith NP , Kooistra T , et al. A human model of asthma exacerbation reveals transcriptional programs and cell circuits specific to allergic asthma. Sci Immunol. 2023;8:eabq6352. 10.1126/sciimmunol.abq6352 PMC10440046 37146132

[11] Iinuma T , Kiuchi M , Hirahara K , et al. Single‐cell immunoprofiling after immunotherapy for allergic rhinitis reveals functional suppression of pathogenic T(H)2 cells and clonal conversion. J Allergy Clin Immunol. 2022;150:850‐860.e5. 10.1016/j.jaci.2022.06.024 35863510

[12] Baysoy A , Bai Z , Satija R , Fan R . The technological landscape and applications of single‐cell multi‐omics. Nat Rev Mol Cell. 2023;24:695‐713. 10.1038/s41580-023-00615-w PMC10242609 37280296

[13] Wu X , Yang X , Dai Y , et al. Single‐cell sequencing to multi‐omics: technologies and applications. Biomark Res. 2024;12:110. 10.1186/s40364-024-00643-4 PMC11438019 39334490

[14] Li DY , Monteiro JP , Zhao Q , Stitziel NO , Quertermous T . Single‐cell studies advance understanding of the genetic and molecular basis of atherosclerosis. Circ Res. 2026;139:e327472. 10.1161/CIRCRESAHA.125.327472 42283082

[15] Eisenbarth D , Wang YA . Glioblastoma heterogeneity at single cell resolution. Oncogene. 2023;42:2155‐2165. 10.1038/s41388-023-02738-y PMC10913075 37277603

[16] He Y , Wei Y , Zhi J , et al. From the brain cell atlas to precision neurology: a review of the application of AI‐driven multi‐omics in brain science. GigaScience. 2026;15:giag075. 10.1093/gigascience/giag075 PMC13354932 42335932

[17] Hellings PW , Klimek L , Cingi C , et al. Non‐allergic rhinitis: position paper of the European Academy of Allergy and Clinical Immunology. Allergy. 2017;72:1657‐1665. 10.1111/all.13200 28474799

[18] Brożek JL , Bousquet J , Agache I , et al. Allergic Rhinitis and its Impact on Asthma (ARIA) guidelines‐2016 revision. J Allergy Clin Immunol. 2017;140:950‐958. 10.1016/j.jaci.2017.03.050 28602936

[19] Huang Y , Wu Y , Han S , et al. Protocol for optimized nasal mucosa sample processing to obtain high‐quality scRNA‐seq and scATAC‐seq data. STAR Protocols. 2024;5:103298. 10.1016/j.xpro.2024.103298 PMC11407050 39244757

[20] McGinnis CS , Murrow LM , Gartner ZJ . DoubletFinder: doublet detection in single‐cell RNA sequencing data using artificial nearest neighbors. Cell Syst. 2019;8:329‐337.e4. 10.1016/j.cels.2019.03.003 PMC6853612 30954475

[21] Korsunsky I , Millard N , Fan J , et al. Fast, sensitive and accurate integration of single‐cell data with Harmony. Nat Meth. 2019;16:1289‐1296. 10.1038/s41592-019-0619-0 PMC6884693 31740819

[22] Yin J , Zheng Y , Huang Z , et al. Chinese immune multi‐omics atlas. Science (New York, NY). 2026;391:eadt3130. 10.1126/science.adt3130 41505528

[23] Eguíluz‐Gracia I , Bosco A , Dollner R , et al. Rapid recruitment of CD14(+) monocytes in experimentally induced allergic rhinitis in human subjectsc. J Allergy Clin Immunol. 2016;137:1872‐1881.e12. 10.1016/j.jaci.2015.11.025 26851967

[24] Nestor CE , Barrenäs F , Wang H , et al. DNA methylation changes separate allergic patients from healthy controls and may reflect altered CD4+ T‐cell population structure. PLoS Genet. 2014;10:e1004059. 10.1371/journal.pgen.1004059 PMC3879208 24391521

[25] Granja JM , Corces MR , Pierce SE , et al. ArchR is a scalable software package for integrative single‐cell chromatin accessibility analysis. Nat Genet. 2021;53:403‐411. 10.1038/s41588-021-00790-6 PMC8012210 33633365

[26] Jin S , Guerrero‐Juarez CF , Zhang L , et al. Inference and analysis of cell‐cell communication using CellChat. Nat Commun. 2021;12:1088. 10.1038/s41467-021-21246-9 PMC7889871 33597522

[27] Jin S , Plikus MV , Nie Q . CellChat for systematic analysis of cell‐cell communication from single‐cell transcriptomics. Nat Prot. 2025;20:180‐219. 10.1038/s41596-024-01045-4 39289562

[28] Kumar N , Mishra B , Athar M , Mukhtar S . Inference of gene regulatory network from single‐cell transcriptomic data using pySCENIC. Methods Mol Biol. 2021;2328:171‐182. 10.1007/978-1-0716-1534-8_10 34251625

[29] Morabito S , Reese F , Rahimzadeh N , Miyoshi E , Swarup V . hdWGCNA identifies co‐expression networks in high‐dimensional transcriptomics data. Cell Rep Meth. 2023; 3:100498. 10.1016/j.crmeth.2023.100498 PMC10326379 37426759

[30] Sakaue S , Kanai M , Tanigawa Y , et al. A cross‐population atlas of genetic associations for 220 human phenotypes. Nat Genet. 2021;53:1415‐1424. 10.1038/s41588-021-00931-x PMC12208603 34594039

[31] Bulik‐Sullivan BK , Loh P‐R , Finucane HK , et al. LD Score regression distinguishes confounding from polygenicity in genome‐wide association studies. Nat Genet. 2015;47:291‐295. 10.1038/ng.3211 PMC4495769 25642630

[32] Voskamp AL , Tak T , Gerdes ML , et al. Inflammatory and tolerogenic myeloid cells determine outcome following human allergen challenge. J Exp Med. 2023;220:e20221111. 10.1084/jem.20221111 PMC10333709 37428185

[33] Carvalheiro T , Horta S , van Roon JAG , et al. Increased frequencies of circulating CXCL10‐, CXCL8‐ and CCL4‐producing monocytes and Siglec‐3‐expressing myeloid dendritic cells in systemic sclerosis patients. Inflamm Res. 2018; 67:169‐177. 10.1007/s00011-017-1106-7 PMC5765192 29127442

[34] Kim HS , Kim YI , Cho JY . ARID3C acts as a regulator of monocyte‐to‐macrophage differentiation interacting with NPM1. J Proteome Res. 2024;23:2882‐2892. 10.1021/acs.jproteome.3c00509 PMC11302414 38231884

[35] Piccolo V , Curina A , Genua M , et al. Opposing macrophage polarization programs show extensive epigenomic and transcriptional cross‐talk. Nat Immunol. 2017;18:530‐540. 10.1038/ni.3710 PMC5524187 28288101

[36] Kanter JE , Bornfeldt KE . Inflammation and diabetes‐accelerated atherosclerosis: myeloid cell mediators. Trends Endocrinol Metab. 2013;24:137‐144. 10.1016/j.tem.2012.10.002 PMC3578033 23153419

[37] Mishra B , Ivashkiv LB . Interferons and epigenetic mechanisms in training, priming and tolerance of monocytes and hematopoietic progenitors. Immunol Rev. 2024;323:257‐275. 10.1111/imr.13330 PMC11102283 38567833

[38] Amrute JM , Perry AM , Anand G , et al. Cell specific peripheral immune responses predict survival in critical COVID‐19 patients. Nat Commun. 2022;13:882. 10.1038/s41467-022-28505-3 PMC8847593 35169146

[39] Tsai MH , Pai LM , Lee CK . Fine‐tuning of type I interferon response by STAT3. Front Immunol. 2019;10:1448. 10.3389/fimmu.2019.01448 PMC6606715 31293595

[40] Lindeboom RGH , Worlock KB , Dratva LM , et al. Human SARS‐CoV‐2 challenge uncovers local and systemic response dynamics. Nature. 2024;631:189‐198. 10.1038/s41586-024-07575-x PMC11222146 38898278

[41] Saichi M , Ladjemi MZ , Korniotis S , et al. Single‐cell RNA sequencing of blood antigen‐presenting cells in severe COVID‐19 reveals multi‐process defects in antiviral immunity. Nat Cell Biol. 2021;23:538‐551. 10.1038/s41556-021-00681-2 33972731

[42] Carpenter S , O'Neill LAJ . From periphery to center stage: 50 years of advancements in innate immunity. Cell. 2024;187:2030‐2051. 10.1016/j.cell.2024.03.036 PMC11060700 38670064

[43] Bernstein JA , Bernstein JS , Makol R , Ward S . Allergic rhinitis: a review. Jama. 2024;331:866. 10.1001/jama.2024.0530 38470381

[44] Zhou S , Zhou C , Wang X , et al. Profiles of immune infiltration in seasonal allergic rhinitis and related genes and pathways. Int Immunopharmacol. 2023;120:110174. 10.1016/j.intimp.2023.110174 37182444

[45] Wang H‐R , Wei S‐Z , Song X‐Y , et al. IL‐1β and allergy: focusing on its role in allergic rhinitis. Mediat Inflamm. 2023;2023:1‐11. 10.1155/2023/1265449 PMC10115535 37091903

[46] Schröder A , Lunding LP , Zissler UM , et al. IL‐37 regulates allergic inflammation by counterbalancing pro‐inflammatory IL‐1 and IL‐33. Allergy. 2022;77:856‐869. 10.1111/all.15072 34460953

[47] Ciprandi G , De Amici M , Murdaca G , et al. Serum interleukin‐17 levels are related to clinical severity in allergic rhinitis. Allergy. 2009; 64:1375‐1378. 10.1111/j.1398-9995.2009.02010.x 19226302

[48] Buggert M , Price DA , Mackay LK , Betts MR . Human circulating and tissue‐resident memory CD8(+) T cells. Nat Immunol. 2023;24:1076‐1086. 10.1038/s41590-023-01538-6 37349380

[49] Moynihan KD , Kumar MP , Sultan H , et al. IL2 targeted to CD8+ T cells promotes robust effector T‐cell responses and potent antitumor immunity. Cancer Discovery. 2024;14:1206‐1225. 10.1158/2159-8290.CD-23-1266 PMC11215410 38563906

[50] Lawson LP , Parameswaran S , Panganiban RA , Constantine GM , Weirauch MT , Kottyan LC . Update on the genetics of allergic diseases. J Allergy Clin Immunol. 2025;155:1738‐1752. 10.1016/j.jaci.2025.03.012 PMC12145254 40139464

[51] Lefevre MA , Vocanson M , Nosbaum A . Role of tissue‐resident memory T cells in the pathophysiology of allergic contact dermatitis. Curr Opin Allergy Clin Immunol. 2021;21:355‐360. 10.1097/ACI.0000000000000763 34155157

[52] Zhang Z , Langenbach M , Sagar S , et al. Efficacy of CTLA‐4 checkpoint therapy is dependent on IL‐21 signaling to mediate cytotoxic reprogramming of PD‐1(+)CD8(+) T cells. Nat Immunol. 2025;26:92‐104. 10.1038/s41590-024-02027-0 PMC12853267 39702858

[53] Fonseca R , Burn TN , Gandolfo LC , et al. Runx3 drives a CD8(+) T cell tissue residency program that is absent in CD4(+) T cells. Nat Immunol. 2022;23:1236‐1245. 10.1038/s41590-022-01273-4 PMC13045866 35882933

[54] Cruz‐Guilloty F , Pipkin ME , Djuretic IM , et al. Runx3 and T‐box proteins cooperate to establish the transcriptional program of effector CTLs. J Exp Med. 2009;206:51‐59. 10.1084/jem.20081242 PMC2626671 19139168

[55] Zhang Y , Lan F , Zhang L . Update on pathomechanisms and treatments in allergic rhinitis. Allergy. 2022;77:3309‐3319. 10.1111/all.15454 35892225

[56] Ogulur I , Mitamura Y , Yazici D , et al. Type 2 immunity in allergic diseases. Cell Mol Immunol. 2025;22:211‐242. 10.1038/s41423-025-01261-2 PMC11868591 39962262

[57] Maldonado A , Angelina A , Cuervo N , Sirvent S , Palomares O . Innate immunity in allergic diseases: novel concepts. J Allergy Clin Immunol. 2026;158:679‐693. 10.1016/j.jaci.2026.06.007 42336292

[58] Tyurin YA , Lissovskaya SA , Fassahov RS , et al. Cytokine profile of patients with allergic rhinitis caused by pollen, mite, and microbial allergen sensitization. J Immunol Res. 2017;2017:3054217. 10.1155/2017/3054217 PMC5646349 29109963

[59] Chen C , Wang J , Pan D , et al. Applications of multi‐omics analysis in human diseases. MedComm. 2023;4:e315. 10.1002/mco2.315 PMC10390758 37533767

[60] Xie C , Wang K , Yang K , et al. Toward precision medicine in COPD: phenotypes, endotypes, biomarkers, and treatable traits. Respir Res. 2025;26:274. 10.1186/s12931-025-03356-w PMC12482825 41024111

[61] Mayassi T , Li C , Segerstolpe Å , et al. Spatially restricted immune and microbiota‐driven adaptation of the gut. Nature. 2024;636:447‐456. 10.1038/s41586-024-08216-z PMC11816900 39567686

[62] Hao Y , Yang Y , Zhao H , et al. Multi‐omics in allergic rhinitis: mechanism dissection and precision medicine. Clin Rev Allergy Immunol. 2025;68:19. 10.1007/s12016-025-09028-3 PMC11836232 39964644

[63] Wang W , Xu Y , Wang L , et al. Single‐cell profiling identifies mechanisms of inflammatory heterogeneity in chronic rhinosinusitis. Nat Immunol. 2022;23:1484‐1494. 10.1038/s41590-022-01312-0 36138182

[64] Tang R , Xia G , Zhu Y , et al. Platelet‐derived serotonin epigenetically programs macrophage alternative activation to orchestrate type 2 airway inflammation. Cell Mol Immunol. 2026. 10.1038/s41423-026-01455-2 PMC13624266 42533042

[65] Tang X‐Z , Kreuk LSM , Cho C , Metzger RJ , Allen CDC . Bronchus‐associated macrophages efficiently capture and present soluble inhaled antigens and are capable of local Th2 cell activation. eLife. 2022;11:e63296 10.1038/s41423-026-01455-2 PMC9560158 36173678

[66] Kooistra T , Saez B , Roche M , et al. Airway basal stem cells are necessary for the maintenance of functional intraepithelial airway macrophages. Cell Rep. 2025;44:115860. 10.1016/j.celrep.2025.115860 PMC12286423 40512621

[67] Schenkel JM , Fraser KA , Beura LK , Pauken KE , Vezys V , Masopust D . T cell memory. Resident memory CD8 T cells trigger protective innate and adaptive immune responses. Science (New York, NY). 2014;346:98‐101. 10.1126/science.1254536 PMC4449618 25170049

[68] Ariotti S , Hogenbirk MA , Dijkgraaf FE , et al. T cell memory. Skin‐resident memory CD8^+^ T cells trigger a state of tissue‐wide pathogen alert. Science (New York, NY). 2014;346:101‐105. 10.1126/science.1254803 25278612

[69] Zhou Y , Wang T , Fan Y , et al. Therapy of allergic rhinitis using ribavirin spray to clear nasal commensal viruses. Signal Transduct Target Ther. 2026;11:249. 10.1038/s41392-026-02744-y PMC13303864 42350364

[70] Guo C‐L , Wang C‐S , Wang Z‐C , et al. Granzyme K(+)CD8(+) T cells interact with fibroblasts to promote neutrophilic inflammation in nasal polyps. Nat Commun. 2024;15:10413. 10.1038/s41467-024-54685-1 PMC11607458 39614076

[71] Lan F , Li J , Miao W , et al. GZMK‐expressing CD8(+) T cells promote recurrent airway inflammatory diseases. Nature. 2025;638:490‐498. 10.1038/s41586-024-08395-9 PMC11821540 39814882

[72] Agrawal K , Ong LC , Monkley S , et al. Allergic sensitization impairs lung resident memory CD8 T‐cell response and virus clearance. J Allergy Clin Immunol. 2022;150:1415‐1426.e9. 10.1016/j.jaci.2022.07.004 35917932

[73] Kwak S , Choi YS , Na HG , et al. Fipronil upregulates inflammatory cytokines and MUC5AC expression in human nasal epithelial cells. Rhinology. 2020;58:66‐73. 10.4193/Rhin19.172 31680128

[74] Simoneau CR , Chen P‐Y , Xing GK , et al. NF‐κB inhibitor alpha controls SARS‐CoV‐2 infection in ACE2‐overexpressing human airway organoids. Sci Rep. 2024;14:15351. 10.1038/s41598-024-66003-2 PMC11222426 38961189

