# Supplementary material for: Single‐cell multi‐omics characterises transcriptional and chromatin accessibility heterogeneity of immune cells in allergic rhinitis
Source: Clin Transl Med. 2026 Oct 13;16(10):e70854. doi: 10.1002/ctm2.70854 (PMC13659270; doi:10.1002/ctm2.70854)
Supplement: Supplementary file 1 [file CTM2-16-e70854-s002.docx]

**Supplementary Figures**

**Supplementary Figure Overview**

**S1**. Study inclusion and baseline characteristics.

**S2**. Overview and multi-omic landscape of immune cell composition.

**S3**. Statistical analysis of immune cell composition.

**S4**. Transcriptional and epigenetic features of myeloid cell subsets.

**S5**. Functional characterization of differentially expressed genes and epigenetic features in Macro_CCL3.

**S6**. Molecular and cell–cell communication features of Macro_CCL3 in AR.

**S7**. Transcriptomic and epigenomic characterization of T cell populations.

**S8**. CD8_Trm exhibits functional heterogeneity in AR.

**S9**. Cell type-specific chromatin accessibility is associated with the genetic risk of allergic diseases.

**S10**. Integrated analysis of clinical traits and T cell gene co-expression networks in allergic rhinitis.

**Supplementary Figure S1**

**Supplementary Figure S1. Study inclusion and baseline characteristics.**

(A) Flowchart of sample inclusion and exclusion. (B) Distribution of age across the NAR, SAR, and PAR groups. Statistical comparisons were performed using Wilcoxon rank-sum tests, with *P* values adjusted using the Benjamini–Hochberg method. (C) Distribution of sex across the NAR, SAR, and PAR groups.

**Supplementary Figure S2**

**Supplementary Figure S2. Overview and multi-omic landscape of immune cell composition.**

(A) UMAP visualization of immune cells from 23 patients, colored by annotated cell subsets (scRNA-seq and scATAC-seq data). (B) UMAP plots from the scRNA-seq dataset, clustered based on gene expression before (left) and after (right) batch correction with Harmony, and colored by disease group. (C) UMAP plots from the scATAC-seq dataset, clustered based on chromatin accessibility before (left) and after (right) batch correction with Harmony, and colored by disease group.

**Supplementary Figure S3**

**Supplementary** **Figure S3. Statistical analysis of immune cell composition.**

(A) Proportion of each cell type in individual samples from the scRNA-seq (left) and scATAC-seq (right) datasets. (B) Cell-type proportions across the NAR, SAR, and PAR groups in the scRNA-seq (top) and scATAC-seq (bottom) datasets. Each dot represents one sample. * indicates *P* < 0.05. Statistical comparisons were performed using Wilcoxon rank-sum tests. (C) Correlation of cell-type proportions between the scRNA-seq and scATAC-seq datasets. Each dot represents one sample. Correlations were assessed using Spearman's rank correlation test.

**Supplementary Figure S4**

**Supplementary Figure S4. Transcriptional and epigenetic features of myeloid cell subsets.**

(A) Dot plot displaying highly expressed marker genes across identified myeloid subsets (size: expression levels; color: expression intensity). (B) Heatmap showing gene activity scores across myeloid cell subsets, with representative signature genes annotated. (C) Cell-type composition of myeloid cell subsets across disease groups, shown for scRNA-seq (left) and scATAC-seq (right). * indicates *P* < 0.05 compared with NAR. (D) Venn diagram showing genes shared between the SAR and NAR groups across differential expression analyses performed using the Wilcoxon rank-sum test and edgeR, respectively. (E) Representative genome browser tracks showing differentially accessible regions (DARs) in CD14_Mono across disease groups. Fold changes relative to NAR and false discovery rate (FDR) values are indicated in the figure. (F) Motif enrichment analysis for the SAR versus NAR and PAR versus NAR comparisons, ranked by the mean differential score. (G) Violin plots showing motif deviation scores across the NAR, SAR, and PAR groups. FDR-adjusted *P* values are shown. (H) Network visualization of CEBPB downstream target genes across myeloid cell populations. Node colors correspond to the cell types shown in the UMAP above. (I) Chromatin accessibility signal tracks highlighting *ACSL1* peak-to-gene links (correlation > 0.5) across myeloid cell types in the region of chromosome 4. Gray shading indicates gene-linked accessible regions. (J) Box plots showing *ACSL1* expression across myeloid cell subsets. Blue boxes indicate significant differences compared with NAR. All statistical comparisons were performed using Wilcoxon rank-sum tests.

**Supplementary Figure S5**

**Supplementary Figure S5. Functional characterization of differentially expressed genes and epigenetic features in Macro_CCL3.**

(A) Volcano plot showing differentially expressed genes (DEGs) in Macro_CCL3 between the SAR and NAR groups. DEGs were defined by Wilcoxon rank-sum test using a cutoff of |log2FC| > 0.25 and FDR < 0.05. (B) Venn diagram showing genes shared between the SAR and NAR groups across differential expression analyses performed using the Wilcoxon rank-sum test and edgeR, respectively. (C) Pathway enrichment analysis of DEGs identified in Macro_CCL3 in the SAR versus NAR comparison. (D) Motif enrichment analysis in Macro_CCL3 for the SAR versus NAR comparison. (E) Dot plot showing the expression levels of IFN-γ-related genes across the indicated cell types. (F) Dot plot showing the expression levels of IFN-γ-related genes in Macro_CCL3 across the NAR, SAR, and PAR groups. Significant differences between SAR and PAR are indicated. (G) Interaction strength of the chemokine signaling pathway between cell subgroups and CD14_Mono or Macro_CCL3 in NAR (left), SAR (middle), and PAR (right). (H) Ligand–receptor pairs associated with chemokine signaling between CD14_Mono or Macro_CCL3 and the indicated cell subgroups. All statistical comparisons were performed using Wilcoxon rank-sum tests.

**Supplementary Figure S6**

**Supplementary Figure S6. Molecular and cell–cell communication features of Macro_CCL3 in AR.**

(A) Volcano plot showing differentially expressed genes (DEGs) in Macro_CCL3 between the PAR and NAR groups. DEGs were defined by Wilcoxon rank-sum test. (B) Pathway enrichment analysis of DEGs identified in Macro_CCL3 in the PAR versus NAR comparison. (C) Transcription factor (TF) motif enrichment analysis for the PAR versus NAR comparison, ranked by mean differential score. (D) Interaction strength of the antigen presentation signaling pathway between cell subgroups and Macro_CCL3 in NAR (left), SAR (middle), and PAR (right). (E) Dot plot showing the expression levels of MHC class II genes across the indicated cell types. (F) Venn diagram showing genes shared between the PAR and NAR groups across differential expression analyses performed using the Wilcoxon rank-sum test and edgeR, respectively. (G) Violin plots showing *HLA-DQA2* expression across the NAR, SAR, and PAR groups. Statistical comparisons were performed using Wilcoxon rank-sum tests. (H) Chromatin accessibility signal tracks highlighting *HLA-DQA2* peak-to-gene links (correlation > 0.5) across groups in the region of chromosome 6. Gray shading indicates gene-linked accessible regions. (I) Scatter plots showing the relationships between *HLA-DQA2* expression levels (log2-transformed pseudobulk expression) and VAS score, total IgE levels, and eosinophil count. Each point represents an individual sample. Spearman's rank correlation coefficients (r) and *P* values are indicated.

**Supplementary Figure S7**

**Supplementary Figure S7. Transcriptomic and epigenomic characterization of T cell populations.**

(A) UMAP plots showing CD4 and CD8 T cells from the scRNA-seq dataset. (B) Dot plot showing highly expressed marker genes across the identified T cell subsets. (C) Heatmap showing gene activity scores across T cell subsets, with representative marker genes annotated. (D) Composition of T cell subsets across the NAR, SAR, and PAR groups. * indicates *P* < 0.05 compared with NAR; # indicates *P* < 0.05 compared with SAR. Each dot represents one sample. Statistical comparisons were performed using Wilcoxon rank-sum tests, with individual samples treated as the statistical units. (E) Heatmap showing differentially expressed genes in tissue CD4 T cells before and after allergen stimulation, based on the GSE49782 dataset. DEGs were identified using limma with an FDR < 0.05 and |log2FC| > 1. (F) Heatmap showing differentially expressed genes in PBMC-derived CD4 T cells before and after allergen stimulation, based on the GSE50223 dataset. DEGs were identified using limma with an FDR < 0.05 and |log2FC| > 1. (G) Motif enrichment analysis of CD4_Tcm cells comparing PAR and SAR, ranked by mean differential score. (H) Heatmap showing the top 10 regulons ranked by normalized regulon specificity score (RSS) in CD4_Tcm cells across the NAR, SAR, and PAR groups.

**Supplementary Figure S8**

**Supplementary Figure S8. CD8_Trm exhibits functional heterogeneity in AR.**

(A–C) Violin plots showing pathway activity scores for T cell migration (A), IL-2 signaling (B), and type II interferon response (C) across the NAR, SAR, and PAR groups. FDR-adjusted P values are shown above the corresponding comparisons. (D) Motif enrichment analysis of CD8_Trm cells comparing the PAR and SAR groups, ranked by mean differential score. (E) Venn diagram showing the overlap of TF motifs identified in the SAR versus NAR and PAR versus NAR comparisons using the CisBP database. All statistical comparisons were performed using two-sided Wilcoxon rank-sum tests.

**Supplementary Figure S9**

**Supplementary Figure S9. Cell type-specific chromatin accessibility is associated with the genetic risk of allergic diseases.**

(A) Heritability enrichment analysis of multiple GWAS traits using cell type-specific chromatin accessibility profiles. Asterisks indicate significant enrichment after multiple-testing correction (adjusted *P* < 0.05; stratified linkage disequilibrium score regression [S-LDSC]). (B) Heritability enrichment of chromatin accessibility peaks across cell types. Enrichment significance was assessed using S-LDSC, with enrichment *P* values derived from the corresponding regression coefficients and standard errors. (C) Heritability enrichment of chromatin accessibility peaks associated with pollinosis and control samples, assessed by coefficient z score.

**Supplementary Figure S10**

**Supplementary Figure S10. Integrated analysis of clinical traits and T cell gene co-expression networks in allergic rhinitis.**

(A) Distribution of clinical parameters, including eosinophil count (EOS count), VAS score, and total IgE levels, across the NAR, SAR, and PAR groups. Statistical comparisons were performed using Wilcoxon rank-sum tests and Benjamini-Hochberg correction. (B) Parameter selection for hdWGCNA, showing the determination of the optimal soft-thresholding power. (C) Weighted gene co-expression network analysis (WGCNA) of T cells, identifying eight gene co-expression modules. (D) Radar plot showing the distribution of the eight gene co-expression modules across different T cell subsets. (E) Gene Ontology (GO) term enrichment analysis of the T cell co-expression modules. (F) Correlation analysis between clinical parameters and T cell co-expression modules. **P* < 0.05, ***P* < 0.01, and ****P* < 0.001. Correlations were assessed using Spearman's rank correlation test.
